# Anthropometry at discharge and risk of relapse in children treated for severe acute malnutrition: a prospective cohort study in rural Nepal

**DOI:** 10.1186/s12937-021-00684-7

**Published:** 2021-04-05

**Authors:** Benjamin Guesdon, Manisha Katwal, Amod Kumar Poudyal, Tusli Ram Bhandari, Emilie Counil, Sujay Nepali

**Affiliations:** 1 Action Against Hunger | Action Contre la Faim (ACF) - France, 14-16 Boulevard Douaumont, 75854 Paris, France; 2 Action Against Hunger | Action Contre la Faim (ACF)- Nepal, Kathmandu, Nepal; 3 Central Department of Public Health, Institute of Medicine (IOM), Tribhuvan University (TU), Kirtipur, Nepal; 4 Department of Public Health, School of Health and Allied Sciences, Pokhara University (PoU), Pokhara, Nepal; 5 Institut national d’études démographiques (INED), F-93322 Aubervilliers, France

**Keywords:** Severe acute malnutrition, Discharge, Relapse, WHZ, MUAC

## Abstract

**Background:**

There is a dearth of evidence on what should be the optimal criteria for discharging children from severe acute malnutrition (SAM) treatment. Programs discharging children while they are still presenting varying levels of weight-for-height (WHZ) or mid-upper-arm circumference (MUAC) deficits, such as those implemented under the current national protocol in Nepal, are opportunities to fill this evidence gap.

**Methods:**

We followed a cohort of children discharged as cured from SAM treatment in Parasi district, Nepal. Relapse as SAM, defined as the occurrence of WHZ<-3 or MUAC < 115 mm or nutritional edema, was investigated through repeated home visits, during six months after discharge. We assessed the contribution of remaining anthropometric deficits at discharge to relapse risk through Cox regressions.

**Results:**

Relapse as SAM during follow-up was observed in 33 % of the cohort (35/108). Being discharged before reaching the internationally recommended criteria was overall associated with a large increase in the risk of relapse (HR = 3.3; *p* = 0.006). Among all anthropometric indicators at discharge, WHZ<-2 led to a three-fold increase in relapse risk (HR = 3.2; *p* = 0.003), while MUAC < 125 mm significantly raised it only in the older children. WHZ<-2 at discharge came up as the only significant predictor of relapse in multivariate analysis (HR = 2.8, *p* = 0.01), even among children with a MUAC ≥ 125 mm. Of note, more than 80 % of the events of relapse as SAM would have been missed if WHZ had not been monitored and used in the definition of relapse.

**Conclusions:**

Our results suggest that the priority for SAM management programs should be to ensure that children reach a high level of WHZ at discharge, at least above or equal to the WHO recommended cut-off. The validity of using a single MUAC cut-off such as 125 mm as a suitable discharge criterion for all age groups is questioned. Further follow-up studies providing a complete assessment of nutritional status at discharge and not based on a restricted MUAC-only definition of relapse as SAM would be urgently needed to set evidence-based discharge criteria. These studies are also required to assess programs currently discounting or omitting WHZ for identification and management of SAM.

## Background

According to the most recent global estimates of child malnutrition, 6.9 % or 47 million children under five years of age were affected by wasting in 2019 [1]. Wasting and severe wasting come with an elevated risk of death [2, 3]. WHO guidance for 6–59 months old children suffering from Severe Acute Malnutrition (SAM), which includes both severe wasting and nutritional edema (kwashiorkor), is that they should be screened, referred, and enrolled into an appropriate therapeutic feeding program [4].

Internationally agreed-upon case definitions for SAM in children of this age include low Weight-for-Height Z-score (WHZ<-3), low Mid-Upper-Arm-Circumference (MUAC < 115 mm), and/or nutritional edema [4, 5]. It is well known that these three criteria identify different children as suffering from SAM, with limited overlap, and that they must be used independently to detect the entirety of the SAM caseload in the population [4, 6]. Since 2013 the World Health Organization (WHO) has recommended that children with SAM should only be discharged from treatment when their WHZ is ≥ − 2 or MUAC is ≥ 125 mm, and they have had no nutritional edema for at least two weeks [5]. Acknowledging the heterogeneity of case definitions, and the resulting variability in anthropometric deficits at admission, WHO further recommended to use the same anthropometric indicator to confirm SAM diagnosis and to assess nutritional recovery under treatment: for instance if MUAC is used to identify that a child has SAM, then MUAC should be used to assess and confirm recovery and decide end of treatment.

This last recommendation on discharge criteria is relying on insufficient evidence. WHO initially called for a stronger evaluation of the validity of MUAC and WHZ as discharge criteria, and the determination of optimal cut-off values [5]; yet to our knowledge this issue has not been further investigated. Notwithstanding, the recent years have been marked by the controversial proposal that programs should stop assessing WHZ, whether for case detection, admission, monitoring or discharge [7]. Although the international normative guidance does not endorse the possibility that SAM treatment programs restrict admission to children with MUAC < 115 mm or nutritional edema [8], this proposal has been increasingly promoted and applied [9, 10]. As opposed to its consequences on targeting and assessing eligibility to treatment, which have been described and discussed elsewhere [6, 11–13], the impacts on discharge have not been analyzed. However, in programs abandoning the assessment of WHZ, MUAC ≥ 125 mm is used as the only restrictive criterion to consider children as cured, irrespective of the WHZ deficits that may be present upon admission. This practice is expected to discharge many children as cured with remaining WHZ deficits [14] and may affect treatment effectiveness to an unknown extent.

Post-treatment outcomes follow-up in children discharged as cured with variable levels of anthropometric deficits is instrumental to fill current evidence gaps about the adequacy of current international recommendation and the extent to which different types of anthropometric deficits at discharge, including WHZ deficits, influence the risk of relapse. In Nepal, severely acutely malnourished children are managed and treated as per the national guideline on Integrated Management of Acute Malnutrition (IMAM) [15]. According to this guidance, all internationally agreed-upon case definitions of SAM (low MUAC or low WHZ or nutritional edema) are eligible for treatment, yet discharge criteria are less stringent than WHO standards: they mainly consist in the observation of a MUAC > 115 mm after a minimum treatment duration of 6 weeks. Many children may thus be discharged from SAM treatment while still presenting anthropometric deficits [14]. The resulting risks have never been assessed. In particular we do not know if (or to what extent) this may predispose to relapse or if instead, the child recovery process would continue after treatment cessation.

We decided to follow children after discharge from SAM treatment in this context to (1) quantify the association between failure to reach WHO-recommended discharge criteria and risk of relapse and (2) identify the respective contribution of different types of anthropometric deficits to increased risk.

## Methods

### Context

Parasi district is located in the Lumbini Province of Nepal, in southern Nepal, with headquarter located in Ramgram. The district has three urban and four rural municipalities. A nutritional survey conducted by the international non-governmental organization Action Against Hunger | Action Contre la Faim (ACF) in Parasi in July 2018 found a very high prevalence of Global Acute Malnutrition, at 25.7 % (21.3–30.1, 95 % CI) and a prevalence of SAM at 3.8 % (2.4–6.1, 95 % CI) [16]. ACF has been providing technical and financial support to the Government of Nepal to roll out inpatient and outpatient SAM management following the IMAM guideline in the district since April 2017. During the study period, inpatient SAM management was provided in Prithivi Chandra Hospital, the district hospital, and outpatient SAM management was provided in 14 health facilities.

### Study design

We conducted a prospective observational study of a cohort of SAM children discharged as cured from the IMAM program. As per the national recommendations of the IMAM protocol, all internationally agreed-upon case definitions of SAM (MUAC < 115mm or WHZ<-3 or nutritional edema) are used to detect and admit SAM children to treatment, yet children are discharged as cured according to the following criteria: a minimum treatment duration of 6 weeks AND MUAC > 115 mm AND no edema for two consecutive visits AND weight gain for the last two consecutive visits AND clinically well and alert. SAM children aged 6 to 59 months at their admission to treatment, and who were discharged as cured as per national recommendations from the 14 health facilities implementing outpatient treatment in Parasi district were the target population. The recruitment period started in July 2019 and was initially scheduled to last one year. The IMAM program registers were screened each week at health facilities to identify children newly discharged as cured as per national protocol. Families were then promptly reached using either direct phone contact or indirect contact through community health workers. Children eligible for the study were all new children discharged as cured (1) for whom a home visit could be planned for a formal recruitment interview with the caretaker at a date close to two weeks after discharge, and (2) for whom the caretaker did not report any plan to leave the district in the next 6 months.

Following the most recent recommendations for assessing and reporting relapse after SAM treatment, follow-up after discharge was implemented through home visits, to minimize loss to follow-up, over 6 months [17, 18]. Seven home visits were planned according to the following schedule: 2 weeks after discharge, 1 month after discharge, and then monthly till 6 months after discharge. Anthropometric measurements were taken at each time point; and questionnaires covering a range of risk factors of undernutrition related to child’s feeding and care practices, the household’s water, hygiene and sanitation conditions, food security and livelihoods were informed. Information on the treatment period, such as admission and discharge characteristics, was collected retrospectively from the health facilities IMAM registers.

Each eligible child contributed to the study until s/he was detected relapse as SAM, was lost to follow-up, or was present at the end of the 6 months of follow-up without relapse. Children displaying relapse as SAM during the follow-up visits were referred to the nearest health facility and managed according to the current IMAM national guideline. They were considered as exiting the study. Caregivers of children detected as suffering from Moderate Acute Malnutrition (MAM), i.e. presenting a -3 ≤ WHZ<-2 or a 115 ≤ MUAC < 125 mm at discharge or follow-up times, received nutrition counselling as per current national guidance yet were kept in the study.

Written informed consent was obtained from the caretakers of all participating children during the recruitment interview. The study was approved by the Institutional Review Committee of the Nepal Health Research Council (NHRC), Nepal.

Of note, the Government of Nepal imposed a nationwide lockdown from the 23rd of March 2020 to prevent the spread of Covid-19. Home visits were then postponed and replaced by phone interviews till lockdown was relaxed partially in Parasi district, on the 20th of May 2020. From this date, home visits resumed only upon explicit approval of the child caretaker; the study staff was wearing adapted personal protective equipment (disposable face masks and gloves) and conducted the measurements and interviews under strict infection prevention and control measures [19]. We however decided to stop recruiting new patients in the cohort from the start of the lockdown period.

### Anthropometric measures

Weight (with a SALTER scale model 135 6 M in the health facilities, and a SECA electronic scale model 874 during home visits), length or height (with a standard UNICEF measuring board), and MUAC (with a standard no stretchable MUAC band) were measured to the nearest 100 g or 1 mm, respectively, as per the most recent recommendations [20, 21]. During the treatment period, anthropometric measurements were taken by health workers with the help of a dedicated IMAM support staff called Nutrition Supervisor. Nutrition Supervisors are contractual staff recruited by Government Health Office but paid by ACF. During home visits, anthropometric measurements were performed by dedicated research officers with the assistance of the caretakers. Great care was provided to ensure the consistency of the measurement procedures and high quality of measure throughout the treatment and the follow-up periods: all measurers were trained anthropometrists who received a refresher training including a standardization test before the start of the study. During the treatment period, the measurements were only taken once and immediately hand written into the IMAM registers. During the home visits, the measurements were taken in duplicate and directly digitalized into a data entry software using pre-programmed quality checks.

To determine age (months), the birth date was either extracted from official documents (e.g. birth certificates) or evaluated by maternal report in reference to a locally adapted seasonal calendar when official documents were unavailable.

### Definition of exposure and outcome variables

In this study, the main exposure variables were WHZ at discharge (further dichotomized as WHZ<-2 vs. WHZ≥-2), MUAC at discharge (further dichotomized as MUAC < 125 mm vs. MUAC ≥ 125 mm), as well as child’s status with regards to the WHO-recommended discharge criteria [5]. Failure to reach WHO-recommended criteria was defined as presenting a WHZ<-2 at discharge when admitted as SAM with a WHZ<-3 and presenting a MUAC < 125 mm when admitted as SAM with a MUAC < 115 mm.

The outcome was relapse as SAM. In agreement with the statement that relapse has been so far inconsistently defined across programs and research [17, 18], we decided to use an inclusive and conservative definition of relapse: we defined relapse as SAM as the occurrence of an event of SAM, i.e. the deterioration of nutritional status from non-SAM to SAM, in children who were initially admitted to SAM treatment and then discharged free of SAM, i.e. WHZ≥-3 and MUAC ≥ 115 and no nutritional edema. In other words, a child discharged as cured was classified relapse as SAM at any time during the 6-months follow-up duration if s/he was presenting a WHZ<-3 or a MUAC < 115 mm or nutritional edema, while not displaying any of these characteristics at the preceding visit. This is an inclusive definition of relapse, as opposed to the idea of distinguishing relapse (in children discharged after reaching WHO-recommended discharge criteria) from regression (in children discharged before reaching WHO-recommended discharge criteria) recently promoted by experts [18]. We argue that all new occurrences of SAM should be counted into the same variable in order to assess the impact of not meeting the currently recommended discharge criteria and further demonstrate their relevance, as requested by the WHO [5]. Relapse as SAM will hereafter be referred to as relapse.

Possible confounders of the association between exposure variables and relapse that were examined were Height-for-Age z-score (HAZ) at discharge (further dichotomized as HAZ<-2 vs. HAZ≥-2), duration of treatment (further dichotomized as < 10 weeks vs. ≥ 10 weeks), age at discharge (further dichotomized as < 24 months vs. ≥ 24 months) and sex [22].

### Statistical analysis

Data were analyzed with Stata (version 13; StataCorp LP, College Station, Texas). Individual Z-scores were computed in reference to the WHO 2006 growth standards [23] using the Stata zscore06 command for WHZ and HAZ [24]. After computation of their admission and discharge characteristics, children who were found to have been admitted to treatment while not being SAM and children who were found to have been discharged from treatment while still SAM were excluded from the analysis.

Discharge characteristics of children identified as relapse or non-relapse during follow-up were compared using Pearson’s chi-squared tests for dichotomous variables and Student’s t-tests for continuous variables.

We estimated the rates of relapse as the number of relapse events by 100 child-months using the Kaplan–Meier approach. For each child contributing to the final analysis, the time to relapse is defined as the time from discharge to the date of a first new episode of SAM, till a maximal duration of observation of 200 days after discharge. Relapse rates are reported over the whole 6 months follow-up as well as during different periods (in the first 3 months period vs. in the following 3 months period).

To assess the influence of anthropometric deficits at discharge, age, sex, and duration of treatment on relapse, survival curves in the two categories of each dichotomized variables were first compared using the log-rank test. We then used multivariate Cox proportional-hazard models to estimate adjusted hazard ratios for low WHZ and low MUAC, including in the model every variable that was significant in the univariate analysis at *p*‐value < 0.2. Interactions between main exposure variables, and with HAZ, age and sex, were also looked for, based on pre-existing hypotheses of effect modifications [22]. We tested the proportional-hazards assumption of the final models based on the Schoenfeld residuals. Hazard ratios with a 95 % confidence interval (CI) that does not cross 1.0 and p-values < 0.05 were regarded as statistically significant.

Acknowledging that a difference in measurement precision between discharge and first follow-up visit may lead to overestimating relapse in children who may rather be in the process of recovery, we performed a sensitivity analysis using a restricted definition of relapse: as an additional criterion, the child had to display a weight loss of more than 200 g since discharge to be classified as relapse at the first follow-up visit. We also assessed the possible impact of the lockdown on our results by censoring all observations at the date of the 23rd of March 2020.

## Results

**Fig. 1:**
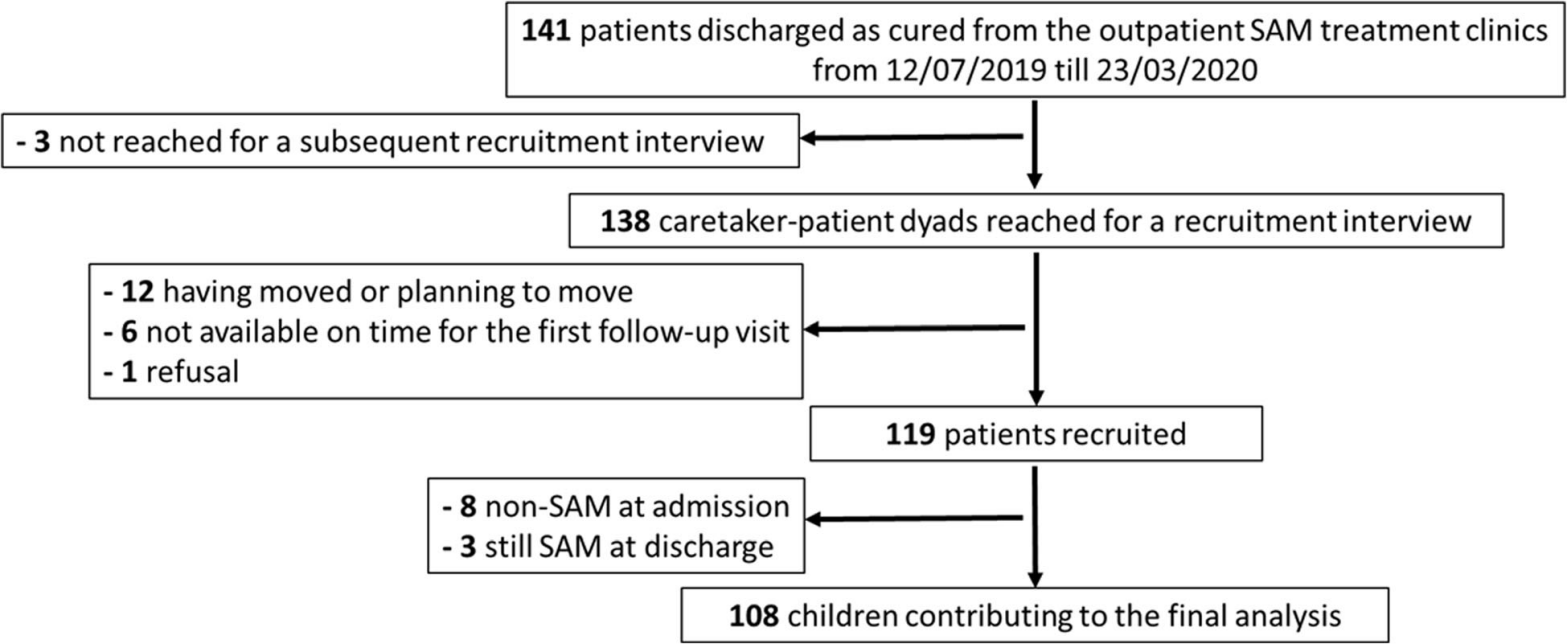
Patients’ flow chart

We recruited 119 children in the cohort and 108 contributed to the final analysis, after the exclusion of 8 patients who were not SAM at admission and 3 who were still SAM at discharge. The study flow chart detailing the recruitment and exclusion process, from the total population of 141 children discharged as cured from the outpatient treatment program between the 12th of July 2019 and the 23rd of March 2020, is shown in Fig. 1. Out of the 108 children included in the final analysis, 102 or 94.4 % of the cohort could be followed till the end of the 6 months follow-up period or till they were detected with a first episode of relapse. 6 children were lost to follow-up, due to internal or cross-border movements and to Covid-19 related restrictions. This resulted in a total of 517.3 child-months of observation available for analysis.

We recorded anthropometric characteristics as well as basic demographic information upon admission, discharge, and relapse (Table 1). Over the 6 months follow-up, 35 children were detected as relapse, i.e. 33.3 % (35/108) of the cohort and 77.1 % (27/35) of these events of relapse occurred during the first three months after discharge. Overall, we observed a rate of relapse of 7.2 by 100 child-months (5.1–10.1; 95 %CI). The incidence rate greatly decreased with time, as shown when comparing the rates between the first 3 months after discharge and the next 3 months (*p* = 0.0003) (Table 2).

**Table 1:** Anthropometric characteristics upon admission, discharge, and relapse till the end of follow-up

Follow-up milestone		Upon Admission (N = 108)	Upon Discharge				Upon Relapse as SAM (N = 35)	Upon last Follow-Up for non-relapsing children (N = 73)
			All children (N = 108)	Children relapsing as SAM (N = 35)	Children not relapsing as SAM (N = 73)	p		
Sex	Girls (%)	53.7	53.7	42.9	58.9	0.12	42.9	58.9
Age (in months)	mean (SD) < 24months (%)	22.0 (13.2) 72.2	24.5 (13.1) 58.3	23.8 (12.8) 60	24.9 (13.4) 58.9	0.70 0,91	26.1 (12.9) 57.1	31.1 (13.5) 31.5
WHZ	mean (SD) <-2 (%)	-3.3 (0.5) 96.3	-1.9 (0.6) 49.1	-2.2 (0.5) 68.6	-1.7 (0.6) 39.7	< 0.001 0.005	-3.2 (0.3) 100	-1.8 (0.7) 49.3
HAZ	mean (SD) <-2 (%)	-2.0 (1.2) 47.7	-2.2 (1.1) 56.5	-2.4 (1.2) 57.1	-2.1 (1.0) 56.2	0.25 0.92	-2.2 (1.1) 54.3	-2.1 (1.0) 53.4
MUAC (mm)	Mean (SD) < 125 mm	120.0 (7.7) 70.4	128.2 (7.4) 33.3	126.0 (6.8) 42.9	129.3 (7.5) 28.8	0.03 0.15	120.7 (5.6) 74.3	131.2 (6.4) 9.6
Type of SAM diagnosis	WHZ-only (%) MUAC-only (%) Both criteria (%)	72.2 7.4 20.4	NA	NA	NA	NA	82.8 8.6 8.6	NA
Type of MAM diagnosis	WHZ-only (%) MUAC-only (%) Both criteria (%) Not MAM (%)	NA	30.6 14.8 18.5 36.1	37.2 11.4 31.4 20.0	27.4 16.4 12.3 43.9	0.30 0.49 0.02 0.02	NA	45.2 5.5 4.1 45.2

*SD* standard deviation; *WHZ* weight-for-height Z-score; *HAZ* height-for-age Z-score; *MUAC* mid-upper arm circumference; *SAM* Severe Acute Malnutrition defined as WHZ<-3 only (WHZ-only) or MUAC < 115 mm only (MUAC-only) or both criteria (no cases of nutritional edema in this setting); *MAM* Moderate Acute Malnutrition, defined as -3 ≤ WHZ<-2 only –WHZ-only) or 115 ≤ MUAC < 125 mm only (MUAC-only) or both criteria

**Table 2:** Incidence rates of relapse as SAM (by 100 child-months)

period	Over the 6 months follow-up		Over the first 3 months		Over the last 3 months	
	Incidence Rate	95 % CI	Incidence Rate	95 % CI	Incidence Rate	95 % CI
Relapse as SAM	7.2	5.1–10.1	10.9	7.5–15.9	2.5	1.0–6.0

**Fig. 2:**
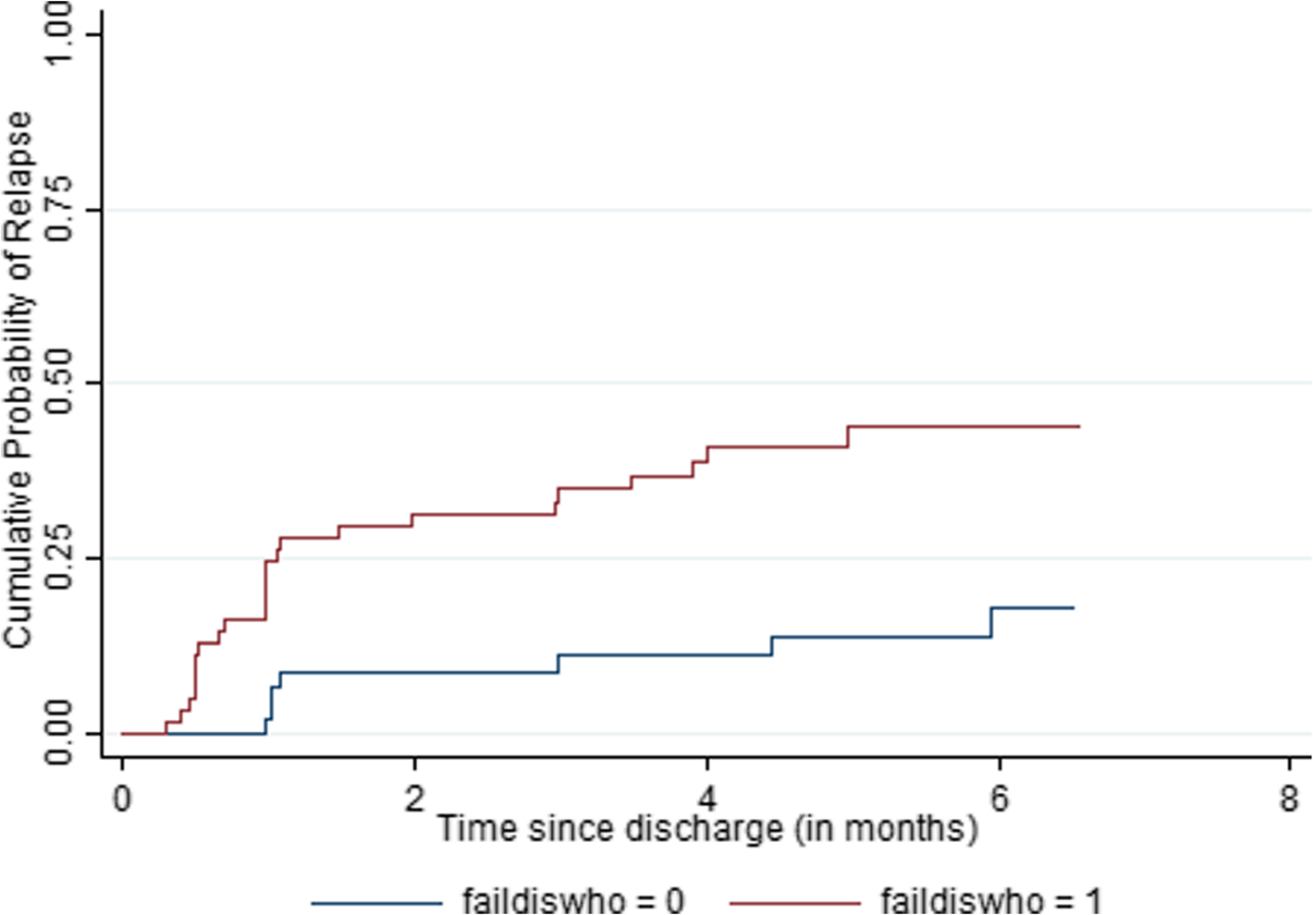
Kaplan-Meier curves of relapse as SAM depending on reach of WHO recommended criteria upon discharge **Legend**: faildiswho = 1 for the group of children who were discharged from treatment before reaching WHO recommended criteria faildiswho = 0 for the group of children who were discharged from treatment before reaching WHO recommended criteria

At discharge, 56.5 % of children failed to reach WHO-recommended discharge criteria, i.e. they failed to reach a WHZ≥-2 when presenting a WHZ<-3 at admission and a MUAC ≥ 125 mm when presenting a MUAC < 115 mm at admission. Failure to reach WHO-recommended criteria at discharge greatly increased the risk of relapse within 6 months after treatment cessation (Fig. 2). Using either univariate or multivariate Cox models adjusted on sex and treatment duration, we assessed that the rate of relapse was increased by a factor greater than 3 (HR = 3.3; 1.4–7.6 95 %CI; *p* = 0.006 in the adjusted model). Among children discharged as cured before reaching WHO-recommended discharge criteria, we observed 42.6 % (26/61) of relapse over the 6-months follow-up, as compared to 19.1 % (9/47) among those reaching WHO-recommended discharge criteria. Anthropometric deficits at discharge classifying children as MAM were, as expected, even more prevalent and observed in 63.9 % of the children (Table 1). Still being MAM at discharge was similarly associated with an increased risk of relapse (HR = 2.7; 1.1–6.8 95 %CI; *p* = 0.03 in the adjusted model).

Beyond these broad categories of combined anthropometric deficits, we then strived to disentangle the respective contribution of each anthropometric parameter at discharge to subsequent risk of relapse (Table 3). The presence of a WHZ<-2 at discharge was the only factor found to significantly increase the risk of relapse, both in univariate and multivariate analysis, while other factors did not, e.g. MUAC deficit (or MUAC < 125 mm), HAZ deficit (or HAZ<-2), younger age (or age < 24months), shorter duration of treatment (or < 10 weeks) and sex. Being discharged as cured with a WHZ<-2 was associated with a 3 times higher risk of relapse after treatment (adjusted HR = 2.8; 1.3–6.2 95 %CI; *p* = 0.01 in the adjusted model). In models using continuous exposure variables, both WHZ and MUAC at discharge were found to affect relapse risk (Table 3). However, again only WHZ remained significantly associated in multivariate models: each increase of 1 Z-score in WHZ at discharge was associated with a division of the risk of relapse by almost 5 times.

**Table 3:** Cox proportional-hazards analysis of the association between anthropometric deficits at discharge and risk of relapse

Exposure variables		Proportion of relapse (%)	Univariate models		Multivariate models	
			Hazard Ratio	p	Hazard Ratio	p
Models with binary exposure variables						
WHZ at discharge	WHZ<-2 WHZ≥-2	45.3 (24/53) 20.0 (11/55)	3.2 Ref.	0.003	2.8 Ref.	0.01
MUAC at discharge	MUAC < 125 mm MUAC ≥ 125 mm	41.7 (15/36) 27.8 (20/72)	1.5 Ref.	0.26	1.4 Ref.	0.32
HAZ at discharge	HAZ<-2 HAZ≥-2	32.8 (20/61) 31.9 (15/47)	1.1 Ref.	0.75	NA	NA
Duration of treatment	< 10 weeks ≥ 10 weeks	22.8 (13/57) 43.1 (22/51)	0.5 Ref	0.051	0.7 Ref.	0.27
Age at discharge	< 24 months ≥ 24 months	32.8 (21/64) 31.8 (14/44)	0.9 Ref.	0.74	NA	NA
Sex	Male Female	40.0 (20/50) 25.9 (15/58)	1.7 Ref.	0.14	1.6 Ref.	0.23
Models with continuous exposure variables						
WHZ at discharge		NA	0.19	< 0.001	0.2	0.001
MUAC at discharge in mm		NA	0.94	0.046	1.0	0.10
HAZ at discharge		NA	0.81	0.24	NA	NA
Duration of treatment in days		NA	1.0	0.10	1.0	0.86
Age at discharge in months		NA	1.0	0.94	NA	NA
Sex	Male Female	40.0 (20/50) 25.9 (15/58)	1.7 Ref.	0.14	1.7	0.19

*WHZ* weight-for-height Z-score; *MUAC* Mid-upper arm circumference; *HAZ* height-for-age Z-score

We found a significant interaction between binary variables of MUAC and age at discharge (p = 0.03). When stratifying the analysis by age groups (Table 4) we found that being discharged with a MUAC < 125 mm was associated with relapse only in children older than 24 months at discharge (HR = 4.1; 1.4–11.8 95 %CI; p = 0.009 in the univariate model). In this age group, both WHZ and MUAC deficits tended to contribute to risk of relapse, with HR of 3.2 and 2.2, respectively, in the multivariate model, although none of them reached statistical significance. On the contrary being discharged with a MUAC below or above the 125 mm cut-off did not affect the risk of relapse in children younger than 24 months at discharge.

**Table 4:** Cox Proportional Hazard models stratified by age groups

Exposure variables		Age < 24 months at discharge				Age ≥ 24 months at discharge			
		Univariate models		Multivariate model		Univariate models		Multivariate model	
		Hazard Ratio	p	Hazard Ratio	p	Hazard Ratio	p	Hazard Ratio	p
WHZ at discharge	WHZ<-2 WHZ≥-2	2.8	0.035	2.8 Ref.	0.037	4.7	0.043	3.3 Ref.	0.13
MUAC at discharge	MUAC < 125 mm MUAC ≥ 125 mm	0.8	0.63	0.8 Ref.	0.69	4.1	0.009	2.2 Ref.	0.17
HAZ at discharge	HAZ<-2 HAZ≥-2	1.5	0.43	NA	NA	0.8	0.66	NA	NA
Duration of treatment	< 10 weeks ≥ 10 weeks	0.8	0.59	NA	NA	0.2	0.032	0.3 Ref	0.10
Sex	Male Female	1.8	0.21	NA	NA	1.6	0.35	NA	NA

*WHZ* weight-for-height Z-score; *MUAC* Mid-upper arm circumference; *HAZ* height-for-age Z-score

We found no significant interaction between WHZ and MUAC deficits at discharge. Consistently, a sub-group analysis conducted in children presenting a MUAC ≥ 125 mm at discharge returned a similarly strong and significant elevation of the risk of relapse associated with being discharged with a WHZ<-2 (HR = 3.0; p = 0.027). We also found no significant interaction between our main exposure variables, i.e. WHZ deficit at discharge or MUAC deficit at discharge, and HAZ at discharge, nor with sex.

In the sensitivity analysis, excluding 3 children who had been previously classified as relapse at the first follow-up visit without displaying a minimum of 200 g weight loss since discharge, or excluding all observations after the start of lockdown lead to very similar results.

Of note, 82.8 % (29/35) of the relapses would not have been detected if we had only used a MUAC < 115 mm or the presence of nutritional edema to define relapse. Even in children initially admitted to treatment with a MUAC < 115, we would have missed 63.6 % (7/11) of the events of relapse, had we been using only MUAC < 115 mm or edema to define relapse as SAM.

## Discussion

### Summary of the main results

Despite being considered as cured as per current national guidance in Nepal, children in our cohort had a high risk of relapse as SAM within 6 months after the end of treatment. Our results show that the current guidance allows discharging a large proportion of children before they reach the WHO-recommended discharge criteria. Among the anthropometric deficits at discharge contributing to the risk of relapse, our results point to a unique role of WHZ deficits. They indicate that discharging children before they reach a WHZ≥-2 dramatically increases their risk of relapse, even when they have already reached a MUAC ≥ 125 mm. Failure to reach a MUAC ≥ 125 mm at discharge was on the contrary not associated with risk of relapse in younger children.

### Novelty of the findings and consistency with other studies

To our knowledge, this is the first study investigating the impact of failure to abide by WHO-recommended discharge criteria for SAM treatment on the risk of relapse, and the respective contributions of remaining MUAC and WHZ deficits at discharge. We are doing so in using an inclusive and conservative definition of relapse as SAM and in following an unselected cohort of children, discharged as cured from SAM treatment with a range of remaining anthropometric deficits. This study also adds to the scarce body of evidence on SAM management coming from South Asia.

So far, the logical hypothesis of an association between lower anthropometric measurements at discharge and negative post-treatment outcomes such as relapse was only supported by a single retrospective study from Burkina Faso [25]. In this study, a significant effect of MUAC < 125 mm at discharge on relapse risk was reported in all children, yet the effect of WHZ was not assessed, MUAC < 125 mm was also the admission criteria, and the definition of relapse was WHZ<-2 or nutritional edema, thereby complicating the interpretation of the results. More recently, the authors of a prospective study in Nigeria reported a high rate of relapse (defined as MUAC < 115 mm or nutritional edema) among children discharged as cured with a MUAC ≥ 125 mm and concluded that this discharge criterion was not adequate to ensure full long-term recovery from SAM [26]. They called for more investigation to understand why the current criteria might be inappropriate in some cases and whether changes or additional criteria could be used to reduce relapse risk. Our results contribute to addressing these questions and to fill the large evidence gap surrounding current international and national recommendations for discharge: they indicate that raising the child’s WHZ to a level at least above or equal to -2 should be the priority to decrease the risk of relapse after treatment, and that the practice of using MUAC ≥ 125 mm as a standalone anthropometric criterion for discharge should be revised.

### WHZ deficits at discharge as a major risk factor of relapse

That WHZ rather than MUAC is the main predictor of relapse in this setting is not surprising. Indeed, analysis of the different types of SAM diagnosis in representative nutritional surveys in Nepal [6] as well as among children admitted to SAM treatment in our cohort shows that WHZ<-3 is the predominant case definition criterion among SAM children and only a minority of SAM children display a MUAC < 115 mm. Since WHZ<-3 is the dominant SAM diagnosis among these children, higher levels of WHZ at discharge may logically reflect a larger recovery during treatment and therefore more sustained protection against nutritional risk and relapse. We believe that a similar result would likely be obtained in all contexts where WHZ<-3 is the predominant anthropometric deficit among SAM cases, which is a prevailing situation in most countries across the world [6, 11]. This is particularly true in the high burden countries of the south-east Asia region, in the Sahel, as well as under acute crisis contexts [27].

### MUAC ≥ 125 mm as an inadequate standalone anthropometric criterion for discharge

Using a 125 mm MUAC cut-off as the only discharge criterion for SAM children admitted with a MUAC < 115 mm has been recommended by WHO for years [5] and is currently part of most national protocols. More recently, it has been pushed forward as a possible standalone criterion in contexts such as the Covid-19 pandemic, where other anthropometric measurements cannot be safely monitored [19]. Besides, the proposal of abandoning the assessment of WHZ for identification and management of SAM has been increasingly promoted and applied in the past years [7, 9, 10]. As a result, it should be expected that many children will be considered as cured while still displaying important levels of WHZ deficits [14]. In revealing a large increase in the risk of relapse associated with WHZ<-2, even in the subgroup of children displaying a MUAC ≥ 125 mm at discharge, our study unveils a potential loss of effectiveness associated with the guidance of using MUAC as a standalone anthropometric criterion for discharge. Obviously, this guidance may be particularly inappropriate for children who are the most likely to reach a high MUAC cut-off with a remaining WHZ deficit, i.e. children admitted to treatment with a WHZ<-3. Programs following the proposal to abandon the assessment of WHZ may be particularly affected by this issue, as they are ignoring the presence of WHZ deficits at admission and do not monitor nor ensure the correction of these deficits. They should thus be applied with caution.

Furthermore, our results reveal that MUAC ≥ 125 mm is not associated with a decreased relapse risk in younger children. The low proportion of children with MUAC < 115 mm at admission in our setting (less than 28 % of our cohort) may have blurred the association of MUAC ≥ 125 mm at discharge and risk of relapse in younger children. However, it should be reminded that according to the WHO growth reference, MUAC is growing with age: while a MUAC of 125 mm corresponds to a MUAC-for-age Z-score (MUACZ) below − 3 in the older children, it corresponds to a MUACZ above − 3 in children below 24 months, and even to a MUACZ above-2 in girls younger than 1 year [28]. In younger children, failure to reach this cut-off may thus not necessarily imply large remaining nutritional deficits and vulnerability. The proper interpretation of MUAC below a fixed cut-off value regarding nutritional status, or its causal relationship with functional outcomes, has long been regarded as problematic because of this age bias [29].

### No apparent increase of risk conveyed by the combination of wasting and stunting

Our study did not find any significant interaction between wasting (i.e. WHZ<-2 or MUAC < 125 mm) and stunting (i.e. HAZ<-2) at discharge on the risk of relapse. This is in apparent contradiction with the recent postulate of a dramatically increased vulnerability for children concurrently wasted and stunted [30]. We however contend that this postulate originates from a single work, which was a re-analysis of former birth cohorts [31] and may not be relevant to children older than 6 months. Recent reanalysis of longitudinal cohort data from Nepal, Senegal, and the Democratic Republic of Congo collected in 1983–1992 indeed reported that SAM children aged 6 months or more who are also stunted neither have a higher case fatality rate nor an elevated mortality risk compared to non-stunted SAM children [3]. Another recent analysis of children aged 6–59 months in the 1983-84 Senegal cohort reported that mortality risk in wasted children did not change significantly based on whether they were stunted or not [32].

### Strengths and limitations

A major strength of this study is the minimal loss to follow-up. This was achieved through the active tracking of patients and the careful organization of the home visits. Besides, the sample of children enrolled in our study consists in a large proportion of the population of children admitted to SAM treatment and discharged as cured in this district of Nepal during the study period. We thus believe that our results are robust and can be generalized to the whole district, and similar contexts in Nepal. However, this study also has some limitations. The first one is our sample size. Although the high proportion of anthropometric deficits at discharge and the high frequency of relapse made it possible to reveal the strong impact of WHZ deficits on the risk of relapse, we may have lacked statistical power to detect more subtle associations, e.g. with duration of treatment or sex, and to detect significant associations in subgroups. Secondly, the recruitment of new patients had to be stopped in March 2020 due to the situation of the Covid-19 pandemic. This decreased our sample size to a large extent. It also prevented us from observing relapse in patients recruited over a whole calendar year and analyzing the impact of seasonality. In particular, we had to stop recruiting new children right before the season of the year expected to correspond to the peak of SAM admission, e.g. higher stress on nutritional security. This may have led to an underestimation of the overall risk of relapse in this context. We also had to reschedule every home visit originally planned during the 2-months lockdown. The observation of any SAM episode occurring during this period was delayed, leading to a possible underestimation of the relapse rate in our study. At the household or community level, the Covid-19 crisis may have modified some contextual risk factors of relapse. A sensitivity analysis excluding all observations after the start of the lockdown yet did not substantially alter our results. Finally, our modest sample size precluded testing the influence of possibly interconnected risk factors of relapse at the household or community levels. We restricted our analysis to the role of discharge criteria, which are amenable to change through the revision of program guidance.

### Recommendations for future studies

Given the current level of uncertainty about the optimal discharge criteria for acute malnutrition management programs, the widespread departure from current WHO recommendations [14], and the growing use of MUAC as the sole indicator for admission and discharge, we believe that similar follow-up studies should urgently be replicated in other contexts, starting with simplified programs abandoning the assessment of WHZ. These studies should investigate the risk of relapse in all children discharged free of SAM from treatment. The first objective should be to document the possibly high rates of relapse induced by alternative discharge practices, such as using only MUAC at a single cut-off of 125 mm to discharge children, and to confirm the role of remaining WHZ deficits. Another objective would be to accumulate data from a range of contexts and profiles of patients to define the optimal discharge criteria for SAM treatment, typically through Receiver Operating Characteristics (ROC) curves analysis. Of note, although being discharged with a WHZ≥-2 appeared as a strong protective factor, there were still 20 % of relapse as SAM among those children. Since WHZ as a continuous variable displayed a strong association with the risk of relapse, the question arises whether a higher WHZ cut-off should be defined to keep relapse risk minimal. Optimal MUAC discharge cut-offs by age group should also be further investigated.

Importantly, such investigations on relapse should fully assess the anthropometry of children upon discharge and during follow-up. They should also use a complete definition rather than a restricted MUAC-only definition of relapse. Not documenting WHZ status at discharge and during follow-up, and using a definition of relapse as SAM restricted to the occurrence of MUAC < 115 mm or nutritional edema, as performed in the past in a range of studies [26, 33–35], would prevent unveiling the strong impact of WHZ deficits at discharge (and maybe of other anthropometric parameters) on post-treatment outcomes, and may seriously underestimate relapse. Had we defined relapse this way, we would indeed have missed more than 80 % of the events of relapse in our study.

## Conclusions

Our results suggest that priority should be given to ensure that the children enrolled in SAM management programs reach a high level of WHZ at discharge, at least above or equal to the WHO-recommended cut-off, and that reaching a MUAC ≥ 125 mm should not be considered as a sufficient discharge criterion. Besides, the validity of using a single MUAC cut-off such as 125 mm as a criterion to end treatment in all age groups should be further investigated. Further follow-up studies providing a complete assessment of nutritional status at discharge and not based on a restricted MUAC-only definition of relapse as SAM, should be performed in a variety of contexts, including in the context of MUAC-only protocols. This is required to assess the current burden of relapse across programs and to build the evidence base for setting discharge criteria that secure sustained recovery and healthy growth.

## Data Availability

The datasets generated and analyzed during the current study are available from the corresponding author on reasonable request and with permission of Action Contre la Faim.
